# Effect of Aquafaba and Almond Milk on the Quality of Gluten-Free Vegan Pancakes: Nutritional and Sensory Evaluation

**DOI:** 10.1007/s11130-025-01311-0

**Published:** 2025-02-22

**Authors:** Gozdenur Tan, Gulcan Ozkan, Ebru Aydin

**Affiliations:** Department of Food Engineering, Faculty of Engineering and Natural Sciences, Suleyman Demirel University, Isparta, Turkey

**Keywords:** Aquafaba, Gluten-free, Vegan, Pancake, Functional food

## Abstract

The rising prevalence of celiac disease, gluten sensitivity, and vegan dietary preferences has increased the demand for gluten-free and plant-based foods. This study investigates the potential of aquafaba, a chickpea cooking by-product, as an egg substitute, combined with almond, coconut, and buckwheat flours to develop gluten-free vegan pancake formulations. Aquafaba was optimized for foaming and emulsifying properties under specific conditions (30 min cooking at 70–80 kPa, 110–115 °C, with a 1:1 chickpea-to-water ratio). Four pancake formulations, including gluten containing pancake (GCP), gluten-containing vegan (GCVP) gluten-free pancake (GFP) and gluten-free vegan pancake (GFVP) were evaluated for physicochemical, nutritional, sensory, and textural properties. The GFVP formulation, containing 10.5% almond, 4% coconut, and 15% buckwheat flours, exhibited 14.38% fat, 8.8% protein, 4.01% ash, and 5.29 g/100 g dietary fiber. These values were significantly higher than GCVP, which had 0.24% fat, 7.75% protein, 2.39% ash, and 0.72 g/100 g dietary fiber. Texture analysis showed that gluten-containing pancakes had superior cohesiveness (0.75) and springiness (0.85), while GFVP demonstrated softer properties with cohesiveness at 0.55 and hardness at 2.7 N. Sensory evaluation revealed high consumer acceptability for GFVP, with competitive scores for flavor and overall preference. Using aquafaba and alternative flours allowed the development of gluten-free, vegan, and sugar-free pancakes with desirable nutritional and sensory attributes. These results highlight aquafaba’s potential in creating sustainable, functional foods tailored to diverse dietary needs. The findings contribute to the advancement of functional, health-focused food development.

**Supplementary Information:**

The online version contains supplementary material available at 10.1007/s11130-025-01311-0.

## Introduction

There is an increasing demand for plant-based and gluten-free products, driven by rising health and environmental concerns. With awareness among consumers regarding overall wellbeing and sustainability, the demand for functional foods that address dietary restrictions is augmenting [1]. Moreover, increasing prevalence of celiac disease and gluten sensitivity, in conjunction with consumer-oriented product development efforts have driven the demand for these healthy products [2].

In addition, meeting these dietary needs promotes more sustainable diets because the high levels of consumption of animal-based foods not only increase the risk for many chronic diseases including heart disease, stroke, and diabetes but also contributes to environmental issues like greenhouse gas emissions, deforestation, and water pollution [3]. Therefore, the development of vegan and gluten-free functional products has become important in order to answer modern consumer demands.

Considering more than 75 billion pancakes are eaten annually across the globe, they help round out a traditional food as well as a means of nutrition [4]. Many of these studies focused on the role pancakes will play in improving whole grain consumption in the U.S [5], while other recent research conducted during COVID-19 pandemic has shown substantial changes to food consumption patterns, further indicating how adaptive and accessible foods like pancakes may be towards changing dietary trends [6].

Aquafaba, a viscous by-product derived from cooking legume seeds such as chickpeas, lentils, or beans, presents a sustainable alternative to traditional egg substitutes in vegan recipes due to its functional properties, including emulsification, foaming, and binding [7]. Aquafaba, has been extensively investigated as a sustainable and functional egg replacer for usage in food formulations. It contains proteins, saponins, and soluble carbohydrates which contribute to its foaming, emulsifying, and gelling capabilities, it has been demonstrated to provide egg white functionality, resulting in an excellent baking ingredient [8]. Studies show that gluten-free and vegan products, like cakes, cookies, and muffins incorporate aquafaba which enhances their texture, moisture retention, and volume [9]. Previous studies have explored various egg substitutes such as flaxseed gel, banana puree and commercial replacers have been researched previously to prepare pancakes. On the other hand, aquafaba provides excellent foaming and emulsification properties similar to egg whites which is important for improving texture, volume, and moisture retention [9]. Compared to alternatives like flaxseed gel and banana puree, aquafaba produces aeration (the addition of air into a mixture) and foam stability (long lasting microbubbles) due to its protein profile; and on top of that, it adds little flavor to the end-product [10, 11]. Using aquafaba in pancake formulations, specifically gluten-free/vegan ones, is however still an area where literature is lacking. Due to the absence of gluten and egg proteins, the typical gluten-free pancakes are of poor textural and sensory quality and require the incorporation of functional substitutes [8]. Yet more systematic studies have neither been performed for the combined use of aquafaba with plant based milk substitutes, *e.g*.*,* almond milk. Almond milk is a widely used calcium alternative because of its effectively free, a lactose-free lower calorie content, and subtle flavor. It is rich in E and D vitamins, so its supplementation improved the moisture, retention and softness of gluten-free and vegan baked goods [12]. This study attempts to bridge the gap by assessing the combination effects of aquafaba and almond milk on gluten-free vegan pancakes physicochemical, nutritional, and sensory properties.Using aquafaba supports global efforts to reduce food waste and boost nutritional value, making it a sustainable choice for both on economy and environment perspective [13]. Pancakes one of the most common breakfast foods in some countries is cooked from eggs, flour, or milk and also can be modified for vegan and gluten-free diets using aquafaba and alternative flours.

This objective of this study is to develop vegan pancakes using aquafaba and gluten-free flours, such as almond, coconut, and buckwheat with an inclusion of erythritol as a low-glycemic sweetener to address the dietary needs of individuals managing diabetes and obesity [14]. Such pancakes have never been assessed in terms of their nutritional and sensory properties; therefore, the goal of this work was to evaluate the physicochemical and sensory characterization of such new pancakes.

Although a variety of gluten-free and/or vegan food products have been investigated, maintaining desirable sensory properties without the use of conventional egg and wheat flour has proven challenging [12, 15]. This study have thus far shown in this research that the combination of aquafaba, as a sustainable egg replacement ingredient with functional gluten-free flours fortified with sponge and texture enhancing psyllium husk fibres can improve respective properties to their post-processing quality characteristics for the first time.

## Materials and Methods

The Materials and Methods section is presented in Supplementary Material 1.

## Results and Discussion

### Sensory Evoluation of Pancakes

The sensory evolution of pancakes plays a major role in consumer acceptability. Pancakes were assessed in this study for four types, i.e., gluten-containing and gluten-free [traditional (with egg) and vegan (egg free)] (Table 1). Statistical analysis indicated no significant differences in taste, odor, texture, or overall preference among the samples (*p* > 0.05). Despite this, there were differences (*p* < 0.05) in color (Supplementary Material 2- Fig. 2), with GCP achieving the highest score. Its traditional look, possibly due to wide adoption of wheat flour and eggs, both of which tend to make light-colored pancakes, may also account for this result. Our results also align with research by Stone et al., [16] demonstrating that consumers prefer products that not only meet health regulations but also mimic sensory profiles in a more traditional way.

**Table 1:** Consumer preference test analysis results

Pancake^*^	Color	Taste	Odor	Texture	Overall Preference
GCP	3.65 ± 0.61^a^	3.58 ± 1.36^a^	3.88 ± 0.88^a^	3.75 ± 0.65^a^	4.08 ± 1.08^a^
GCVP	2.77 ± 0.70^b^	2.75 ± 1.22^a^	3.25 ± 0.75^a^	3.29 ± 0.91^a^	3.33 ± 0.98^a^
GFP	3.54 ± 0.68^ab^	3.29 ± 0.81^a^	3.67 ± 1.05^a^	3.44 ± 0.71^a^	3.50 ± 1.00^a^
GFVP	2.79 ± 0.91^b^	3.33 ± 0.94^a^	3.92 ± 0.67^a^	3.58 ± 0.70^a^	4.00 ± 1.04^a^

Note: Tukey’s multiple comparison test applied for each characteristic. Different superscript letters in the same column indicate significant differences (*p* < 0.05). *Gluten-containing Pancake (GCP): Gluten-containing pancake produced using wheat flour and cow’s milk; Gluten-Free Pancake (GFP): Gluten-free pancake produced using buckwheat, coconut, and almond flours with cow’s milk; Gluten-containing Vegan Pancake (GCVP): Gluten-containing vegan pancake produced using wheat flour with almond milk; Gluten-Free Vegan Pancake (GFVP): Gluten-free vegan pancake produced using buckwheat, coconut, and almond flours with almond milk

Gluten-free formulations (GFP and GFVP) were darker in color than pancakes containing gluten (GCP and GCVP). This disparity is probably due to the Maillard reaction, because almond and coconut flours are rich in amino acids and sugars and promote browning during the cooking process [17]. Moreover, the buckwheat flour used naturally had more pigment than the wheat flour which led to decreased brightness of these formulations [18].

Despite being darker in color and the observation of no significant differences in taste, odor, or texture, GFVP presented a similar overall preference score, which may indicate that it is a suitable candidate as an additive to gluten-free products with acceptable sensory quality, even though it achieved a darker colour [19]. In contrast, GCVP had the lowest overall preference scores which could be related to the combination of aquafaba and wheat flour that may cause some modification of sensory profile because aquafaba’s limited ability to mimic the sensory contributions of eggs [20].

## Physicochemical Properties and Proximate Composition

The amount of moisture may determine the product’s quality, shelf life, and processability. Moisture content of pancake formulations that were significantly different were shown (*p* < 0.05). In terms of moisture, gluten formulations (GCP: 44.31% and GCVP: 44.09%) were not statistically different among them and remained with the highest moisture contents and gluten-free formulations (GFVP: 37.87% and GFP: 37.26%) also clustered with each other and exhibited lower moisture contents. The increased moisture in GCP and GCVP is probably attributed to water-binding ability of gluten and egg. The lesser moisture retention capacity of buckwheat flour, on the contrary may have played a part in the lower moisture levels in the gluten-free formulations [21, 22]. The increased moisture retention of almond flour compared with coconut flour could also help explain the slight differences between GFVP and GFP [23].

**Table 2:** Proximate composition of pancake samples

Parameter	GCP^*^	GCVP^*^	GFVP^*^	GFP^*^
Moisture (%)	44.31 ± 0.03^a^	44.09 ± 0.11^a^	37.87 ± 0.08^b^	37.26 ± 0.64^b^
Ash (%)	2.55 ± 0.020^c^	2.39 ± 0.031^b^	4.01 ± 0.004^a^	3.94 ± 0.008^a^
pH	7.14 ± 0.050^a^	6.89 ± 0.061^b^	6.74 ± 0.001^b^	6.53 ± 0.089^c^
Protein (%)	8.81 ± 0.04^d^	5.13 ± 0.01^c^	7.75 ± 0.04^a^	10.38 ± 0.04^b^
Fat (%)	4.06 ± 0.06^c^	0.23 ± 0.04^d^	14.38 ± 0.13^b^	16.95 ± 1.053^a^
Carbohydrate (%)	39.24 ± 0.12^b^	47.44 ± 0.05^a^	30.71 ± 0.03^c^	26.51 ± 1.79^d^
Sugar (%)	6.10 ± 0.04^b^	0.71 ± 0.02^d^	3.40 ± 0.03^c^	9.21 ± 0.01^a^
Salt (%)	0.44 ± 0.02^b^	0.39 ± 0.02^b^	0.53 ± 0.00^a^	0.50 ± 0.00^a^
Energy (kcal)	228.71 ± 0.15^c^	212.37 ± 0.19^d^	283.22 ± 1.21^b^	300.07 ± 2.49^a^
TF (g/100 g)	1.04 ± 0.04^c^	0.72 ± 0.04^d^	5.29 ± 0.05^a^	4.96 ± 0.07^b^
TISF (g/100 g)	1.04 ± 0.04^c^	0.72 ± 0.04^d^	3.38 ± 0.05^a^	4.96 ± 0.07^b^
TSF (g/100 g)	< 0.65	< 0.65	1.91 ± 0.05^a^	< 0.65

Tukey’s multiple comparison test applied. Different superscript letters in the same row indicate significant differences (*p* < 0.05). TF: Total Soluble and Insoluble Dietary Fiber, TSF: Soluble Dietary Fiber, TISF: Insoluble Dietary Fiber. ^*^Abbreviations are defined under Table 1

Formulations differed significantly in the analysis of ash content (*p* < 0.05). The ash content in gluten-free formulations (GFVP: 4.01% and GFP: 3.94%) significantly exceeded than gluten-containing formulations (GCP: 2.55% and GCVP: 2.39%). This is due to the higher mineral content of alternative flours like almond, coconut, and buckwheat flours [24]. Ash content in GCVP is found to be lower, which could be due to the interaction of the ingredients replacing the cow’s milk and eggs that boasts more minerals than almond milk and aquafaba [19].

The GFVP showed the highest total fiber (TF: 5.29 g/100 g) and insoluble dietary fiber (TISF: 3.38 g/100 g) levels compared to GFP (TF: 4.96 g/100 g, TISF: 3.38 g/100 g). There was significant difference (*p* < 0.05) in TF content between these two formulations, proving higher individual contribution of almond, coconut, and buckwheat flours in GFVP, compared to GFP. Due to the sources of high-fiber flours in gluten-containing formulations (GCP: 1.04 g/100 g and GCVP: 0.72 g/100 g), they showed lower TF values. Importantly, GFVP also had the highest TSF value (1.91 g/100 g), whereas TSF values for all the remaining formulations were below the detection limit (< 0.65 g/100 g), indicating the undisputed higher soluble fiber content of GFVP [25]. This suggests that GFVP has much higher TSF content than any other formulations which corresponds to the findings of Hassan [25] who noted the much higher TSF content in gluten free bakery applications (coconut and almond flour) as rich source of soluble dietary fiber. The TISF content in GFVP and GFP is due to the addition of buckwheat flour, which is rich in insoluble fibers [26]. Moreover, gluten-free formulations with almond, coconut, and buckwheat flour show increased soluble and insoluble dietary fiber content [27].

Having aquafaba, which contributes a limited amount of dietary fiber, was poor in GCVP, while aquafaba as part of the GFVP composition, showed a significantly different TF content [19]. This difference reflects the much larger contribution of flours made from almonds, coconuts, and buckwheats in GFVP compared to the contributions of wheat flour and almond milk in GCVP. These findings are consistent with those reported by Dinnah et al., [28] which found that almond and coconut flours significantly increase dietary fiber content in gluten-free products. The second supporting factor was the high insoluble fiber content of buckwheat flour [18].

There were significant differences in protein content among formulations (*p* < 0.05). The protein content was highest in GFP (10.38 g/100 g), followed by GCP (8.81 g/100 g), GFVP (7.75 g/100 g), and lowest in GCVP (5.13 g/100 g). The enhanced GCP protein content compared to GFVP can be attributed to the fact that egg, which has a high-quality protein content of 9.7–11% [20], was added in GCP. GFVP, on the other side, is based on aquafaba which contributes a lot less protein (0.95–1.5%) compared to that of eggs, resulting in lower overall protein levels. Moreover, gluten as protein network is found in wheat flour in GFCP and not in GFVP so that could also boost the protein content in GCP [12].

GFP and GFVP’s higher protein content is due to the protein-rich nature of almond, coconut, and buckwheat flours [29]. GFVP’s use of aquafaba as an egg replacer, however, does reduce its overall protein content compared to GCP. These findings shed light on the importance of eggs and gluten for protein enrichment in conventional formulations and the demand for protein-rich substitutes for vegan-based products.

The sugar level of GFP formulation (9.21%) was found to be at the highest as compared to all other respective formulations due to sugars made in nature by coconut and almond flours along with lactose present in cow milk [26]. This low sugar content in the GFVP (3.40%) and GCVP formulations is probably because of the replacement of cow’s milk by almond milk, with much lower concentration of sugar [30]. These results highlight the importance of ingredient selection in influencing nutrition composition, potentially allowing for both carbohydrate-rich or low-sugar dietary choices.

The carbohydrate and sugar content analysis, presented in Table 2, revealed statistically significant differences among the formulations (*p* < 0.05). GCVP contained the most carbohydrates (47.44%) because of the inclusion of wheat flour and aquafaba. The contribution to the total carbohydrates was mainly due to the presence of high starch content in wheat flour. This formulation also exhibited the lowest levels of sugar (0.71%) despite its high carbohydrate content which can be attributed to the low sugars present in aquafaba and almond milk, a body of evidence suggesting that aquafaba contributes negligible sugars as an alternative egg replacer [31]. In contrast, the GFP, which contained coconut, almond, and buckwheat flours, showed the lowest carbohydrate content (26.51%). This decrease probably results from increased fat and protein contents of these flours replacing part of the carbohydrates. This observation is consistent with findings from Buchel et al., [32], who noted similar reductions in carbohydrate content with almond and coconut flours added to baked products [32]. The GFP formulation (9.21%) had the highest sugars, as it was mainly related to the sugar content of almond and coconut flours and lactose from cow’s milk [26]. The lower sugar content in the GFVP (3.40%) and GCVP formulations is likely due to the substitution of cow’s milk with almond milk, which contains significantly less sugar [30]. The results of this study highlight the impact of ingredient choice in determining the nutritional characteristics that can allow a high-carbohydrate versus low-sugar dietary approach.

### Color Analysis Results of Pancakes

The color analysis revealed significant differences in brightness (L^*^), redness (a^*^), and yellowness (b^*^) across pancake formulations (Supplemantary Material 2- Table 1). For top surface, GCVP displayed a significantly higher brightness (L^*^ = 80.74) than GCP (61.44), GFP (65.77) and GFVP (68.03) (*p* < 0.05). This is consistent with Hassan [25], where almond milk creates a lighter color than darker flours like buckwheat and coconut. GCP and GFVP had statistically equivalent brightness although the latter contained darker flours. This similarity is likely due to the effectiveness of cow’s milk and eggs in GCP; both ingredients promote the signature golden-brown coloring while masking the darkness associated with Maillard reactions [27].

The GCVP had the highest hair brightness on the bottom surface (L^*^= 85.13), followed by GFP (77.95), GCP (72.69) and GFVP (68.99). The lighter brightness of GFVP is due to the naturally darker and more colored flours used such as almond, buckwheat, and coconut which are in agreement with literature [18]. In terms of chromatic values, GCP exhibited the highest values for yellowness (b^*^= 29.39) due to the presence of eggs and cow’s milk, and GCVP demonstrated the lowest redness (a^*^= 5.50), probably owing to the use of fewer components that stimulate Maillard reactions.

For the inner surface, GCP had the highest brightness (L^*^= 96.78), followed by all other formulations (*p* < 0.05). The L^*^ of GFVP was the lowest (L^*^= 75.72), indicating the contribution of down colored flours, such as buckwheat. Due to its lighter coloring and the absence of darker flours, GCVP and GFP had relative brightness that was not significantly different (L* = 83.58 and 83.52). In terms of redness (a), GFVP (7.08) had the highest value, probably affected by the buckwheat flour [26] reddish pigmentation, whereas, in terms of yellowness (b), the highest value (25.44) was attributed to the GCP, as expected due to the egg and cow’s milk contributions. GFVP had the greatest ΔE from GCP on all surfaces, especially on the inner surface (ΔE = 22.64.) This shows how much the choice of ingredients affects the color of the surface and inside.

### The Texture Analysis of the Pancake Formulations

The texture analysis of the pancake formulations reveals distinct differences influenced by gluten and animal-based proteins (Supplemantary Material 2- Table 3). The GCP displayed higher values for hardness, cohesiveness, springiness, gumminess, and chewiness, aligning with literature where gluten enhances structural properties [12]. In contrast, the GFP exhibited lower values in these attributes due to the absence of gluten’s viscoelastic network, consistent with findings for gluten-free baked products [2]. The GCVP maintained moderate textural properties due to gluten’s presence, though almond milk substitution slightly reduced cohesiveness and springiness [15]. Lastly, the GFVP demonstrated the lowest values across all texture attributes, reflecting the combined absence of gluten and animal proteins, leading to a softer, less elastic texture profile as supported by literature on gluten-free vegan alternatives [2, 12].

Radar chart of sensory evaluation results confirming the above texture findings (Supplementary Material 2- Fig. 1). Gluten-free samples (GFCP and GFVP) yielded lower overall acceptability, taste and texture parameters indicating gluten structure functionality contributed to a firmer cohesive texture profile that was in agreement with the objective texture analysis scoring higher on GCP and GCVP (gluten containing sample products). In particular, the GCP sample ranked highest in all sensory attributes including flavour and aroma traits consistent with the incorporation of gluten and other animal-based ingredients which correlated with increased chewiness, springiness and gumminess observed during texture analysis. In comparison, gluten-free vegan samples (GFP and GFVP) received a lower score on texture and overall acceptability, which was in accordance with the softer and low elasticity texture evident from physical analysis. This integrated sensory and texture analysis illustrates the effect of ingredient selection on both objective textural measurements and subjective consumer perceptions, highlighting the importance of formulation selection in meeting texture expectations in gluten-free and vegan products.

The observed synergistic action in the present study corroborate previous studies on aquafaba foaming and emulsifying properties [20] and almond milk’s role in improving moisture and texture [12]. To disentangle the respective influences of each formulation on texture, nutritional quality, and sensory attributes, future work should investigate single-variable trials where only aquafaba or non-dairy milk is present.

## Conclusion

This study evaluated the impact of gluten-free and vegan ingredients, including almond, coconut, and buckwheat flours, aquafaba, and almond milk, on the physicochemical and nutritional properties of pancakes. Findings showed that selection of ingredients had a powerful impact on protein, fat, carbohydrate, sugar, salt and energy content of the end products.

Higher amounts of fat content in gluten-free formulations (GFP and GFVP) were due to the almond and coconut flours; thus, resulting in a nutrient-dense profile. GCP, though had a higher protein content than GFVP, which may be attributed to the inclusion of eggs and cow’s milk that the GFVP was devoid of, thereby increasing the protein level significantly. GCVP was a vegan formula with the lowest fat and energy content, representing a low-calorie plant-based option, with a sugar content lower than the other formulas, although catering to the interest of a specific consumer market. As you can see, the differences in salt content were due to replacing higher-sodium cow’s milk and eggs with lower-sodium almond milk and aquafaba.

GCP had been presumed to have a darker surface color due to the presence of alternative flours as a result of color analysis, yet these samples also had a similar darker appearance together with gluten-free samples (GFP and GFVP). For GCVP and GFP it could be noted statistically similarity of brightness (L^*^), which further proves the action of almond milk in lightening formulations. Texture analysis indicated that formulations containing gluten possessed greater values of hardness, cohesiveness, springiness, gumminess, and chewiness, leading to firmer and more elastic textures. Gluten-free and vegan formulations, on the other hand, were more soft and less cohesive, highlighting the textural roles of gluten and animal proteins.

In conclusion, alternative gluten-free and vegan ingredients allow its flexibility in the formulation of the pancakes, covering different diets needs, from high-protein and high-energy to low-fat and low sugar products. Further studies, based on single variable analyses, would thus be capable of revealing the independent roles of aquafaba and almond milk on gluten-free systems.

## Electronic Supplementary Material

Below is the link to the electronic supplementary material.

Supplementary Material 1

Supplementary Material 2

## Data Availability

No datasets were generated or analysed during the current study.
