# Supplementary material for: Effect of Aquafaba and Almond Milk on the Quality of Gluten-Free Vegan Pancakes: Nutritional and Sensory Evaluation
Source: Plant Foods Hum Nutr. 2025 Feb 22;80(1):72. doi: 10.1007/s11130-025-01311-0 (PMC11846757; doi:10.1007/s11130-025-01311-0)
Supplement: Supplementary file 1 — Supplementary Material 1 [file 11130_2025_1311_MOESM1_ESM.docx]

**Supplemantary Material 1**

**Effect of Aquafaba and Almond Milk on the Quality of Gluten-Free Vegan Pancakes: Nutritional and Sensory Evaluation**

**Plant Foods for Human Nutrition**

**Gozdenur Tan^1^, Gulcan Ozkan^1^, Ebru Aydin^1^**

^1^Department of Food Engineering, Faculty of Engineering and Natural Sciences, Suleyman Demirel University, Isparta, Turkey

Corresponding author: [ebruaydin@sdu.edu.tr](mailto:ebruaydin@sdu.edu.tr), <https://orcid.org/0000-0002-5625-040X>

Material and Methods

Material

The pancake mixture was prepared with the following ingredients: special-purpose wheat flour (Erişler Gıda Sanayi ve Ticaret A.Ş., Istanbul, Turkiye), coconut and almond flours (İngro Gıda Bilişim Pazarlama, Karaman, Turkiye), buckwheat flour (İpek Değirmen Gıda Sanayi, Aksaray, Turkiye), powdered sugar (Konya Şeker Sanayi ve Ticaret A.Ş., Konya), salt (Rafine Billur Tuz Sanayi A.Ş., İzmir), 3.5% fat cow's milk (Pınat Süt Mamülleri Sanayi A.Ş., İzmir, Turkiye), unsweetened almond milk (Fomilk Bitkisel Gıda Üretim A.Ş., Istanbul), baking powder (Naturus Gıda, İzmir, Turkiye), and erythritol (Genuss Plus, Germany). Chickpeas and eggs were purchased from a local market (Yayla, Ankara, Turkiye). Additives, including xanthan and guar gum (Tito, İzmir, Turkiye) were bought online.

Methods

Aquafaba production

The investigations involved foaming and emulsifying properties of chickpea water in relation to egg replacement for cake formulations within this study. Aquafaba was prepared based on previously publish method [1]. First, washing of chickpeas was done to remove dirt, dust and other objects. To obtain the chickpea water, 100 g of chickpeas were soaked in 400 ml of water at 4°C for a period of 16 h. After soaking, the chickpeas were strained and rinsed with distilled water. The chickpeas were then cooked with the pressure cooker at 70–80 kPa and 115–118°C with the addition of 100 ml water for a period of 30 min. Chickpea water at 1:1 ratio resulted in high foam volume and formation found in preliminary trials which aided the texture of cake formulations. Previous researches reported that the foaming and emulsifying properties of chickpea water improved at a pH range between 4.5 and 5.0 [2]. Despite the lower foaming and emulsifying properties of chickpea proteins around their isoelectric point (~ pH 4.5–4.6), flours, fats, and soluble compounds present in pancake batter may stabilize these properties providing aquafaba with adequate functionality for food applications [17, 18]. To achieve the optimum foaming performance, in this study, citric acid was added to adjust the pH of chickpea water at 4.6. The aquafaba was then stored at -18°C until used in cake formulations.

**Formulation Development of Pancake**

The main goal of this study was to formulate a variety of dietary-specific pancake formulations (i.e., gluten-free, vegan, and sugar-free) that preserve important sensory attributes (e.g., taste, texture, and appearance). Preliminary trials were conducted to optimize the formulations, followed by sensory evaluations to ensure high consumer acceptability [5].

Gluten-containing and gluten-free vegan pancakes were developed at the Functional Foods Laboratory, Department of Food Engineering, Süleyman Demirel University. Four different formulations of pancakes were created (1) gluten-containing pancake mixture (GCP), (2) gluten-containing vegan pancake mixture (GCVP), (3) gluten-free pancake mixture (GFP) and (4) gluten-free vegan pancake mixture (GFVP); formulated to promote the balance of texture, taste and nutritional composition found in each formulation respectively (Table 1). The formulations containing gluten were prepared with conventional wheat flour, while the gluten-free versions were based on a mixture of buckwheat, coconut and almond flours. All vegan formulations used aquafaba (egg substitute from chickpeas) as eggs substitute and all vegan formulations contained unsweetened almond milk as a dairy substitute.

Baking powder was utilized as the leavening agent to achieve the desired lightness and fluffiness in the pancakes. The present study investigated the impact of baking powder to create a co-leavening effect with aquafaba, a material that mainly behaves as an emulsifier and binder and does not provide much aeration. Similar strategies in gluten-free and vegan formulations have shown that the use of baking powder guarantees consistent leavening, texture, and product volume [6, 7]. Xanthan gum and guar gum were used in equal ratio to improve textural integrity of the product and stability of the batter. Erythritol was used as the sweetener to minimize caloric intake while preserving sweetness, in line with the goal of producing sugar-free pancakes. A small amount of salt was added to enhance the overall flavor profile.

**Table 1.** Ingredients for Pancake Formulations (100 g).

| **Ingredients** | **GCP (g)^*^** | **GFP (g) ^*^** | **GCVP (g) ^*^** | **GFVP (g) ^*^** |
| --- | --- | --- | --- | --- |
| Wheat Flour | 28.97 | - | 30.32 | - |
| Buckwheat Flour | - | 14.49 | - | 15.16 |
| Almond Flour | - | 10.62 | - | 11.12 |
| Coconut Flour | - | 3.86 | - | 4.04 |
| Cow's Milk | 38.62 | 38.62 | - | - |
| Almond Milk | - | - | 40.42 | 40.42 |
| Egg | 19.31 | 19.31 | - | - |
| Aquafaba | - | - | 14.55 | 14.55 |
| Erythritol | 11.74 | 11.74 | 12.29 | 12.29 |
| Xanthan Gum | 0.096 | 0.096 | 0.10 | 0.10 |
| Guar Gum | 0.096 | 0.096 | 0.10 | 0.10 |
| Baking Powder | 1.043 | 1.043 | 1.09 | 1.09 |
| Salt | 0.125 | 0.125 | 0.125 | 0.125 |
| Lemon | - | - | 1.01 | 1.01 |

*Gluten-containing Pancake (GCP): Gluten-containing pancake produced using wheat flour and cow’s milk; Gluten-Free Pancake (GFP): Gluten-free pancake produced using buckwheat, coconut, and almond flours with cow’s milk; Gluten-containing Vegan Pancake (GCVP): Gluten-containing vegan pancake produced using wheat flour with almond milk; Gluten-Free Vegan Pancake (GFVP): Gluten-free vegan pancake produced using buckwheat, coconut, and almond flours with almond milk.

In the preliminary stages, cooking parameters such as temperature and time were adjusted to ensure a golden-brown colour and that all pancakes were cooked consistently. Originally, aquafaba took 2–3 minutes of whipping in an electric mixer on medium speed to create a light foam. That ensured that it had the foaming and aerating qualities that egg whites have. Then, the whipped aquafaba was carefully folded in with almond milk and other liquid ingredients to prevent deflation. Once the liquid mixture was blended, the dry ingredients (flours, gums, baking powder, salt, erythritol, and lemon) were added and mixed gently until the batter was smooth [8]. Each pancake was cooked on a preheated electric hotplate using a 1/8 cup (30 mL) measure of batter. Pancakes were cooked for 3-8 minutes, flipping once to ensure even browning. Each batch produced approximately 8-9 pancakes, each weighing approximately 20 grams. It was essential that all the samples had the same sensory characteristics; thus size and cooking process were standardized.

**Sensory Analysis**

The overall acceptability, texture attributes (hardness, chewiness), flavor, stickiness and colour of pancakes prepared from different ratios of flours with salt and water were evaluated on a 5 point hedonic scale with1 representing "extremely bad" to 5 being the "most excellent". This sensory evaluation was conducted by 21 trained panelists from Süleyman Demirel University, including faculty members, graduate students, and administrative personnel.

efore the tastings, panelists underwent a short training course that covered tasting techniques (i.e., rinsing with water in between samples) and standardized protocols for chewing and swallowing. Each pancake sample was presented on a white plate to panelists with three random, single-digit numbers written on the plates to mask their identities.

**Physicochemical Analysis**

The standard gravimetric method was used to determine the moisture content in pancakes. Based on the Association of Official Analytical Chemists AOAC, (2005) method, samples were placed in a drying oven (FN-500, Nuve, Ankara, Turkey) at 105°C overnight [9]. Methods for determining total fat, protein, salt and ash content were performed according to AOAC[10]. The pancakes sugar content was measured with AOAC 923.09 method AOAC [10]. The extraction of dietary fiber was performed according to AOAC method 991.43, and the determination of pH values followed AOAC method 981.12 [9].

Energy content was calculated according to the method described based on the AOAC 2000 (method 996.06) for energy values were assigned as 4 kcal/g for carbohydrates and proteins, and 9 kcal/g for fats. The total energy (kcal) was calculated using Equation 1:

Total Energy (kcal)=(Carbohydrates×4)+(Protein×4)+(Fat×9)

**Texture Analysis**

Through texture profile analysis method, the baked pancake samples were analyzed for their texture characteristics [11]. Texture profile analysis was performed using a TA-XT Texture Analyzer (TA-XT Plus, Texture Stable Micro Systems, Godalming, UK). Due to optimal pancake cooking and preparation conditions. The texture profile was analyzed with 2 cycles of compression: a flat-ended cylindrical aluminum probe, 75 mm in diameter, was used. Five pancake strips were placed on the analyzer surface. Each pancake was compressed to 50% of the initial height at a compression speed of 1 mm/s as described by Cho et al., [12]. Quantitative parameters from the time–force curve, including hardness, adhesiveness, springiness, cohesiveness and resilience were also measured. Measurements repeated at least five times and results are presented as mean ± standard error. These measurements were then used for the characterization of textural properties of pancakes.

**Statistical Analysis**

One-way analysis of variance (ANOVA) and Tukeys post hoc test were performed to investigate the statistical significance (p < 0.05) among the pancake formulations. Statistical analysis differences were determined between sequences using Tukey's multiple comparison test. All experiments were carried out in duplicate and all statistical analyses were performed using Minitab software.

References

[1] He Y, Meda V, Reaney MJT, Mustafa R (2021) Aquafaba, a new plant-based rheological additive for food applications. Trends Food Sci Technol, 111:27–42.

[2] Lafarga T, Villaró S, Bobo G, Aguiló-Aguayo I (2019) Optimisation of the pH and boiling conditions needed to obtain improved foaming and emulsifying properties of chickpea aquafaba using a response surface methodology. Int J Gastron Food Sci, 18:100177. DOI: 10.1016/j.ijgfs.2019.100177

[3] He Y, Meda V, Reaney MJT, Mustafa R (2021) Aquafaba, a new plant-based rheological additive for food applications. Trends Food Sci Technol, 111:27–42.

[4] Buhl TF, Christensen CH, Hammershøj M (2019) Aquafaba as an egg white substitute in food foams and emulsions: Protein composition and functional behavior. Food Hydrocoll, 96:354–364.

[5] Aydin E et al. (2023) A new approach for the development and optimization of gluten-free noodles using flours from byproducts of cold-pressed okra and pumpkin seeds. Foods, 12(10):2018.

[6] Ozcelik MM, Aydin S, Aydin E, Ozkan G (2024) Preserving nutrient content in red cabbage juice powder via foam‐mat hybrid microwave drying: application in fortified functional pancakes. Food Sci Nutr, 12(2):1340–1355. DOI: 10.1002/fsn3.3137

[7] AOAC (2005) Official method of analysis. Association of Official Analytical Chemists.

[8] AOAC (2000) Association of official analytical chemists. Official Methods of Analysis, 12.

[9] Larrosa V, Lorenzo G, Zaritzky N, Califano A (2016) Improvement of the texture and quality of cooked gluten-free pasta. LWT, 70:96–103. DOI: 10.1016/j.lwt.2016.02.039

[10] Cho E et al. (2019) Influence of physicochemical characteristics of flour on pancake quality attributes. J Food Sci Technol, 56:1349–1359.
