# Supplementary material for: Effect of Aquafaba and Almond Milk on the Quality of Gluten-Free Vegan Pancakes: Nutritional and Sensory Evaluation
Source: Plant Foods Hum Nutr. 2025 Feb 22;80(1):72. doi: 10.1007/s11130-025-01311-0 (PMC11846757; doi:10.1007/s11130-025-01311-0)
Supplement: Supplementary file 2 — Supplementary Material 2 [file 11130_2025_1311_MOESM2_ESM.docx]

**Supplemantary Material 2**

**Effect of Aquafaba and Almond Milk on the Quality of Gluten-Free Vegan Pancakes: Nutritional and Sensory Evaluation**

**Plant Foods for Human Nutrition**

**Gozdenur Tan^1^, Gulcan Ozkan^1^, Ebru Aydin^1^**

^1^Department of Food Engineering, Faculty of Engineering and Natural Sciences, Suleyman Demirel University, Isparta, Turkey

Corresponding author: [ebruaydin@sdu.edu.tr](mailto:ebruaydin@sdu.edu.tr), <https://orcid.org/0000-0002-5625-040X>

**Table 1.** Color parametersa of pancakes

| Top Surface | L* | a* | b* | ΔE* |
| --- | --- | --- | --- | --- |
| GCP | 61,44±2.08^b^ | 14,93±1.72^a^ | 20,22±2.09^b^ | 0,00 ±0,00 |
| GCVP | 80,74±3.47^a^ | 11,60±1.33^a^ | 28,06±2.35^a^ | 20.67±0.82^a^ |
| GFP | 65,77±5.89^b^ | 13,63±1.75^a^ | 21.33±4.01^ab^ | 4.87±0.18^b^ |
| GFVP | 68,03±2.34^b^ | 11,80±1.43^a^ | 20,25±3.07^b^ | 7.01±0.21^b^ |
| Bottom Surface | | | | |
| GCP | 72,69±7.58^ab^ | 12,94±1.86^a^ | 29,39±7.05^a^ | 0,00 ±0,00 |
| GCVP | 85,13±1.64^a^ | 5,50±2.56^b^ | 23,04±5.44^a^ | 15.03±0.76^a^ |
| GFP | 77,95±.2.61^ab^ | 13,44±1.25^a^ | 30,83±0.91^a^ | 5.73±0.19^b^ |
| GFVP | 68,99±5.77^b^ | 13,55±0.68^a^ | 22,43±2.71^a^ | 7.34±0.22^b^ |
| Inner Surface | | | | |
| GCP | 96.78±1.52^a^ | 2.04±0.01^b^ | 25.44±1.57^a^ | 0,00 ±0,00 |
| GCVP | 83.58±1.6^b^ | 0.72±0.74^b^ | 17.04±0.92^b^ | 15.42±0.77^a^ |
| GFP | 83.52±1.05^b^ | 6.21±0.37^a^ | 19.48±2.12^b^ | 15.36±0.76^a^ |
| GFVP | 75.72±0.29^c^ | 7.08±0.74^a^ | 17.33±1.65^b^ | 22.64±0.89^a^ |

## Note: Tukey’s multiple comparison test applied. Columns read top to bottom show statistically significant differences (p<0.05). "±" symbol indicates standard deviation while letter notation (a, b) expresses statistical significance between values with different letters in the same column. L* values represent lightness, with lower values indicating darker colors and higher values lighter colors. a* values are negative for green tones and positive for red tones. b* values are negative for blue tones and positive for yellow tones.

## Gluten-containing Pancake (GCP): Gluten-containing pancake produced using wheat flour and cow’s milk; Gluten-Free Pancake (GFP): Gluten-free pancake produced using buckwheat, coconut, and almond flours with cow’s milk; Gluten-containing Vegan Pancake (GCVP): Gluten-containing vegan pancake produced using wheat flour with almond milk; Gluten-Free Vegan Pancake (GFVP): Gluten-free vegan pancake produced using buckwheat, coconut, and almond flours with almond milk.

**Table 2**. Texture analysis of pancake formulations (n=5).

| **Sample^*^** | **Hardness (g)** | **Cohesiveness** | **Springiness** | **Gumminess** | **Chewiness (N)** |
| --- | --- | --- | --- | --- | --- |
| **GCP** | 4.8±1.02^a^ | 0.75±0.04 ^a^ | 0.85±0.08 ^a^ | 4.0±1.01 ^a^ | 4.5±0.98 ^a^ |
| **GFP** | 3.2±0.44^b^ | 0.60±0.02 ^b^ | 0.65±0.01 ^b^ | 3.0±0.92 ^b^ | 3.3±0.66 ^b^ |
| **GCVP** | 4.1±0.98^c^ | 0.70±0.02 ^c^ | 0.80±0.09 ^c^ | 3.5±0.86 ^c^ | 4.0±0.48 ^c^ |
| **GFCP** | 2.7±0.54^d^ | 0.55±0.03 ^d^ | 0.60±0.01 ^d^ | 2.5±0.93 ^d^ | 2.8±0.18 ^d^ |

Note: Tukey’s multiple comparison test applied for each characteristic. Different superscript letters in the same row indicate significant differences (p<0.05). ^*^ Abbreviations are defined under Table 1.


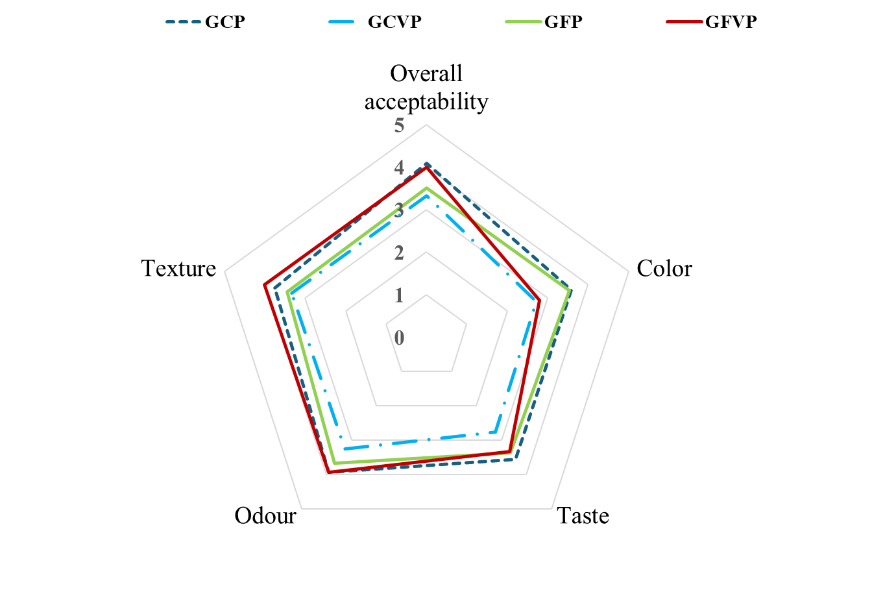


Figure 1. Radar chart of Sensory Evaluation Results for Gluten/Gluten-Free Vegan Pancake Samples. Abbreviations are defined under Table 1.


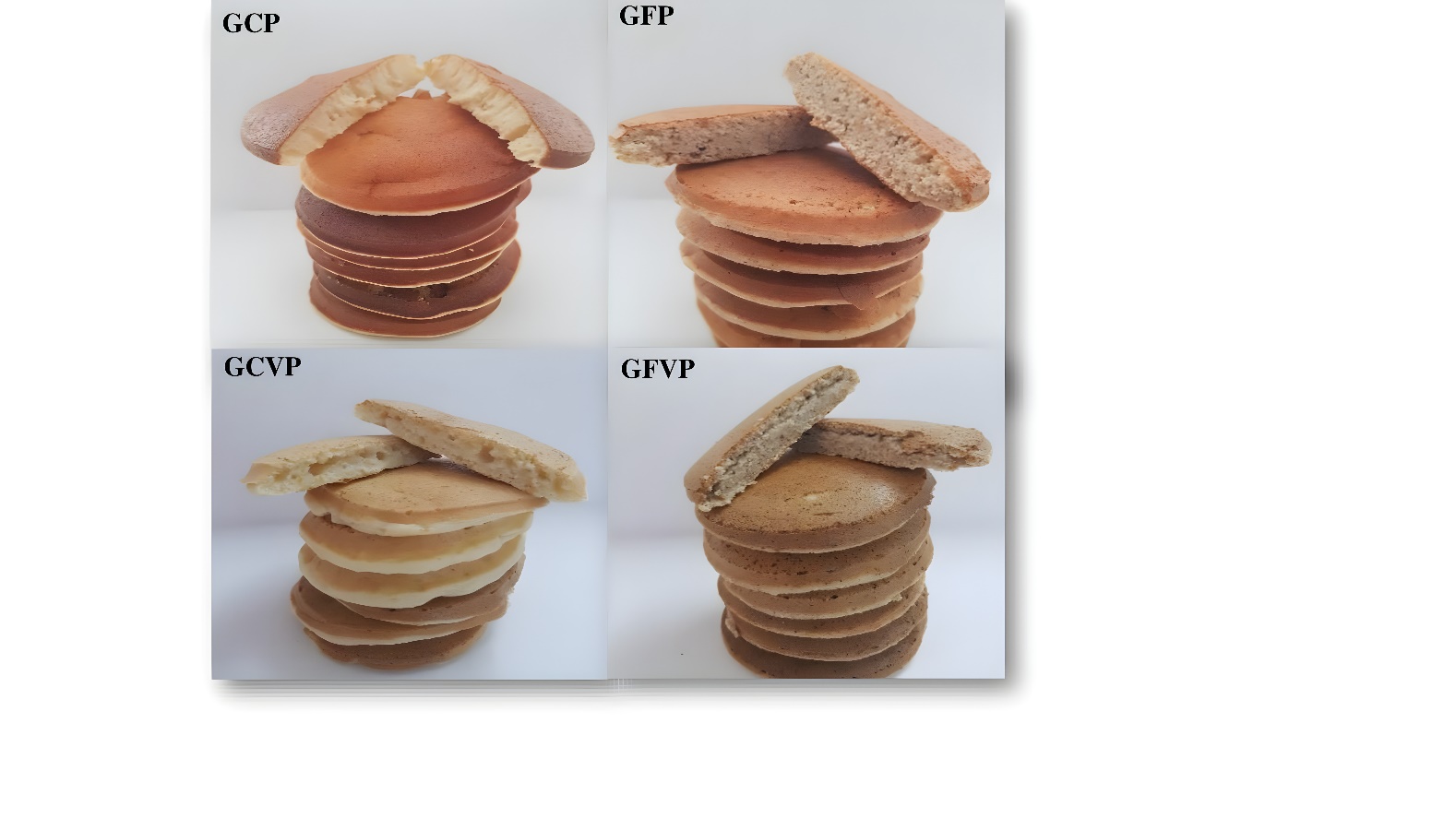


**Figure 2.** The pictures of pancakes. Abbreviations are defined under Table 1.
